# Three-Dimensional Imaging of Terahertz Circular SAR with Sparse Linear Array

**DOI:** 10.3390/s18082477

**Published:** 2018-07-31

**Authors:** Jubo Hao, Jin Li, Yiming Pi

**Affiliations:** School of Information and Communication Engineering, University of Electronic Science and Technology of China, Chengdu 611731, China; jubohao@163.com (J.H.); ympi@uestc.edu.cn (Y.P.)

**Keywords:** terahertz, circular SAR, sparse linear sensor array, sparse recovery, 3D imaging

## Abstract

Due to the non-contact detection ability of radar and the harmlessness of terahertz waves to the human body, three-dimensional (3D) imaging using terahertz synthetic aperture radar (SAR) is an efficient method of security detection in public areas. To achieve high-resolution and all aspect imaging, circular trajectory movement of radar and linear sensor array along the height direction were used in this study. However, the short wavelength of terahertz waves makes it practically impossible for the hardware to satisfy the half-wavelength spacing condition to avoid grating lobes. To solve this problem, a sparse linear array model based on the equivalent phase center principle was established. With the designed imaging geometry and corresponding echo signal model, a 3D imaging algorithm was derived. Firstly, the phase-preserving algorithm was adopted to obtain the 2D image of the ground plane for each sensor. Secondly, the sparse recovery method was applied to accomplish the scattering coefficient reconstruction along the height direction. After reconstruction of all the range-azimuth cells was accomplished, the final 3D image was obtained. Numerical simulations and experiments using terahertz radar were performed. The imaging results verify the effectiveness of the 3D imaging algorithm for the proposed model and validate the feasibility of terahertz radar applied in security detection.

## 1. Introduction

Terrorist attacks have increased substantially in recent years on a global scale, and they pose a great threat to public safety. Security detection in public areas is an important approach to guard against attacks in advance [1,2]. Compared with detection methods like metal detectors and manual checking, three-dimensional (3D) imaging techniques can provide more precise information, rendering automatic detection and identification possible. Recently, 3D synthetic aperture radar (SAR) imaging has attracted increasing research interest because of its ability to obtain the real spatial position and scattering properties of the targets  of interest [3,4,5,6,7]. On the other hand, terahertz (THz) waves have proved superior in their ability to penetrate clothes with inappreciable harm to the human body, and it is thereby suitable for concealed weapon detection. Besides, terahertz waves have a short wavelength and are able to generate wide bandwidths, which can achieve high-resolution imaging [8,9,10]. Therefore, 3D imaging using terahertz radar is a promising method in security detection.

Among various kinds of 3D imaging patterns, circular SAR (CSAR) is popular for its 3D imaging ability and full $360^{{}^{\circ}}$ observation with a single pass [11,12,13]. CSAR 3D imaging needs a pitch angle to realize resolution in the height direction, which may limit its application in some cases. Besides, because of the pitch angle, resolutions between range and height directions are coupled, which makes it difficult to improve both resolutions simultaneously. To address this issue, much research has been conducted by scholars, including studies on multipass SAR, linear array SAR, etc. [14,15,16,17,18]. In [16], holographic SAR (HoloSAR) was applied to form 3D images, where it combines the height reconstruction with the 2D imaging of multipass CSAR. Compared with single-pass CSAR 3D imaging, HoloSAR can reduce sidelobes and provide more height details. However, HoloSAR may face the nonuniform height-sampling problem, which reduces the image quality. The sensor array is another choice for 3D SAR imaging, where the array distributes along the needed direction to provide a sampling aperture. However, to satisfy the half-wavelength spacing condition to avoid grating lobes, a large number of sensors are needed, which leads to a complicated and costly system, especially for terahertz radar. Therefore, we need to reduce the number of sensors to make the radar system realizable.

A sparse array was considered for 3D SAR imaging, however, the traditional Fourier-based imaging method may be invalid in this condition [19,20]. Fortunately, the targets of interest in security detection behave with spatial sparsity generally, thus, the sparse recovery method can be employed to form the image. Among the multiple sparse recovery algorithms, sparse Bayesian learning (SBL) has superiority to deterministic algorithms due to its characteristic of incorporating prior knowledge [21,22]. Without knowing the signal sparsity, SBL estimates the distribution parameters and the posterior with iteration, which can obtain more stable solutions. In Ref. [23], SBL was applied to through-wall imaging with missing data measurements, and in [24], a local structural SBL was adopted to form ISAR images of maneuvering targets with compressively sampled data. The desired results were obtained in both of the studies. Therefore, SBL was used for height reconstruction in this study due to its favorable performance.

In this work, the imaging geometry of CSAR with sparse linear sensor array was first established, and the echo signal model in the terahertz band was developed. Then, a 2D ground plane imaging procedure using phase-preserving algorithm was deduced for each sensor. After that, the phase features of the 2D images were analyzed, and reconstruction along the height direction was transformed into a sparse recovery problem. After completing the reconstructions of all the range-azimuth cells, the 3D image was obtained. The rest of this paper is organized as follows. In Section 2, the 3D imaging geometry with sparse linear sensor array is provided and the imaging procedure is described. In Section 3, simulations and experiments are presented and analyzed to demonstrate the effectiveness of the 3D imaging method for the proposed imaging model. Conclusions are given in the last section.

## 2. 3D Imaging Methodology

### 2.1. Imaging Geometry

**Figure 1:**
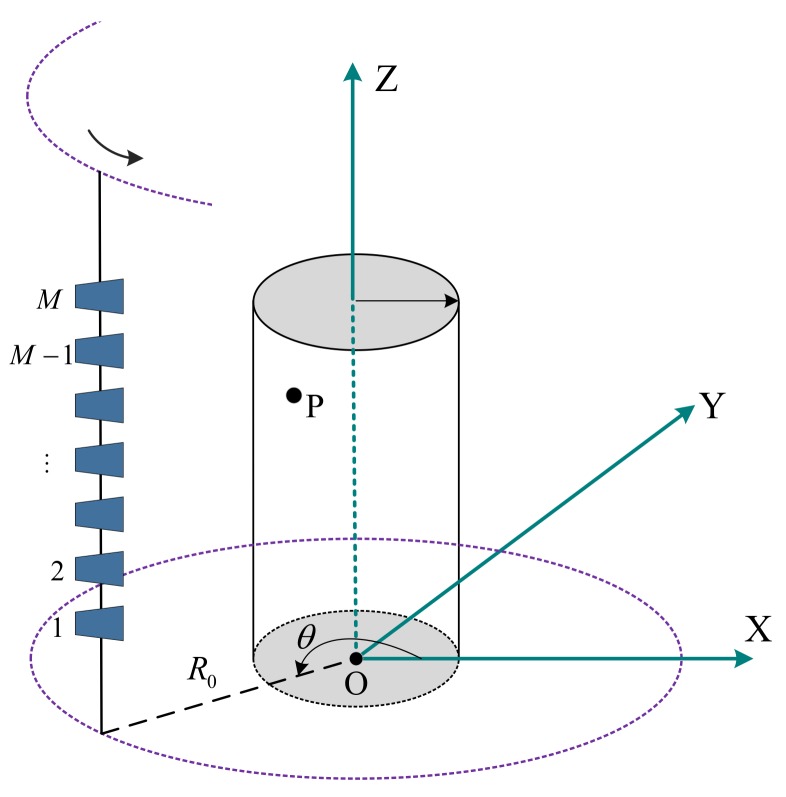
Imaging geometry of circular synthetic aperture radar (CSAR) with sparse linear sensor array.

There are several different transmitting-receiving models for a sensor array, such as single-transmitter multiple-receiver (STMR) and multiple-transmitter multiple-receiver (MTMR). Based on the equivalent phase center principle, it can be considered equivalent to the model that treats each sensor transmits signal and receives echo independently. The specific transmitting-receiving pattern design is not discussed in this paper, and the equivalent distribution of the sensor array is presented directly. The imaging geometry of CSAR with a sparse linear sensor array is shown in Figure 1, where the radar system consists of a vertical sparse linear array operating at a terahertz band. The sensor array spreads parallel to the *Z*-axis and moves along a circular trajectory with radius $R_{0}$. Assuming that there are a total of *M* equivalent sensors whose *Z*-axis coordinates are ${\bar{v}}_{m},m=1,\ldots ,M$, respectively, when the radar system moves to the azimuth angle θ, the instantaneous coordinate of the equivalent sensor *m* is $\left(R_{0}\cos\theta ,R_{0}\sin\theta ,{\bar{v}}_{m}\right)$. It is assumed that the target scattering is isotropic and the sensors can illuminate the region of interest during the entire sampling time.

In general, a target can be approximated as a sum of several independent and non-directional scattering centers in SAR imaging [8]. Therefore, the echo of the targets can be expressed as the superposition of echoes of all scattering centers. Considering a general scattering center *P* with coordinate $\left(x_{P},y_{P},z_{P}\right)$, which is plotted in Figure 1, the instantaneous distance from the equivalent sensor *m* to the scattering center is given as

(1) $$r_{m}\left(\theta \right)=\sqrt{{\left(R_{0}\cos\theta -x_{P}\right)}^{2}+{\left(R_{0}\sin\theta -y_{P}\right)}^{2}+{\left({\bar{v}}_{m}-z_{P}\right)}^{2}}$$

With the assumption that the target is in the far-field, the fourth and higher-order terms can be neglected for the Taylor series expansion of the cosine term. The distance expression in Equation (1) can be approximated as

(2) $$r_{m}\left(\theta \right)=2\left[R_{cm}-\frac{{\bar{v}}_{m}z_{P}}{R_{cm}}-\frac{R_{0}}{R_{cm}}\left(x_{P}\cos\theta +y_{P}\sin\theta \right)\right]$$

where $R_{cm}=\sqrt{R_{0}^{2}+{\bar{v}}_{m}^{2}}$ is the distance from the equivalent sensor *m* to the origin O.

### 2.2. 2D Phase-Preserving Imaging Method

For terahertz radar, broadband can be realized, and the pulsed linear frequency modulated (LFM) signal using the dechirp-on-receive technique is appropriate for high-resolution imaging [6,8]. The transmitted radar signal is presented as

(3) $$s\left(\tau \right)=rect\left(\frac{\tau }{T}\right)\exp\left(j2\pi f_{0}\tau +j\pi \gamma \tau^{2}\right)$$

where τ is the fast time, *T* is the pulse duration, $f_{0}$ is the center frequency, and γ is the modulated rate. The rectangular window is defined as $rect\left(\tau /T\right)=1$ for −T/2<τ<T/2 and =0 otherwise.

For scattering center *P*, the echo received by sensor *m* is expressed as

(4) $$s_{b}\left(\tau ,\theta ;m\right)=\rho_{P}rect\left(\frac{\tau -\tau_{d}}{T}\right)\exp\left[j2\pi f_{0}\left(\tau -\tau_{d}\right)+j\pi \gamma {\left(\tau -\tau_{d}\right)}^{2}\right]$$

where $\rho_{P}$ is the scattering coefficient of *P*, and $\tau_{d}=r_{m}\left(\theta \right)/c$ is time delay, where *c* is light speed.

For broadband signal, the matched filtering (MF) method will need a high sampling rate to satisfy the Nyquist sampling criterion, which could increase the data size remarkably. Nevertheless, the dechirp technique is able to overcome this difficulty and is used extensively in terahertz radar signal processing. The dechirped signal of Equation (4) is

(5) $$s_{if}\left(\tau ,\theta ;m\right)=\rho_{P}rect\left(\frac{\tau -\tau_{d}}{T}\right)\cdot \exp\left\{-j\frac{2\pi }{c}\left(f_{0}+\gamma \tau \right)\cdot r_{m}\left(\theta \right)\right\}\cdot \exp\left\{j\pi \gamma {\left[\frac{r_{m}\left(\theta \right)}{c}\right]}^{2}\right\}$$

In Equation (5), the second exponential term is the residual video phase (RVP), and it can be removed before the next steps [25].

Next, the signal will be transformed into wavenumber domain. Let $K=2\pi \left(f_{0}+\gamma \tau \right)/c$, $K_{0}=2\pi f_{0}/c$, and ΔK=2πγT/c represent the signal wavenumber, center wavenumber, and bandwidth wavenumber, respectively. Substituting (2) into (5), we get

(6) $$s_{if}\left(K,\theta ;\varphi_{m}\right)=\rho_{P}rect\left(\frac{K-K_{0}}{\Delta K}\right)\cdot \exp\left\{-j2K\left[R_{cm}-z_{P}\sin\varphi_{m}-\cos\varphi_{m}\left(x_{P}\cos\theta +y_{P}\sin\theta \right)\right]\right\}$$

where $\varphi_{m}$ represents the pitch angle between the equivalent sensor and the XOY plane and satisfies $\cos\varphi_{m}=R_{0}/R_{cm}$, $\sin\varphi_{m}={\bar{v}}_{m}/R_{cm}$.

In Equation (6), for a determined equivalent sensor *m*, the first phase term can be compensated with compensating factor $H_{c}=\exp\left(j2KR_{cm}\right)$. The wavenumber of *K* can be decomposed as follows

(7) $$\left\{\begin{array}{c} K_{x}=-2K\cos\varphi_{m}\cos\theta \\ K_{y}=-2K\cos\varphi_{m}\sin\theta \end{array}\right.$$

By substituting (7) into (6), the signal can be represented as

(8) $$s_{if}\left(K_{x},K_{y};\varphi_{m}\right)=\;\rho_{P}rect\left(\frac{K-K_{0}}{\Delta K}\right)\cdot \exp\left(j2Kz_{P}\sin\varphi_{m}\right)\cdot \exp\left\{jK_{x}x_{P}+jK_{y}y_{P}\right\}$$

As mentioned in Section 2.1, the echo of the entire region of interest is the superposition of all the scattering centers. Thus, the radar echo received by the *m*th sensor is expressed as

(9) $$s_{a}\left(K_{x},K_{y};\varphi_{m}\right)=\;\int \int \int \;\rho \left(x,y,z\right)\cdot rect\left(\frac{K-K_{0}}{\Delta K}\right)\cdot \exp\left(j2Kz\sin\varphi_{m}\right)\cdot \exp\left\{jK_{x}x+jK_{y}y\right\}dxdydz$$

where ρ(x,y,z) is the scattering coefficient of a scattering center located at (x,y,z).

The triple integral in Equation (9) can be decomposed into two parts, one is a double integral about dxdy, the other is single integral about dz. Since the window function in (9) has no direct impact on the phase analysis, it can be neglected for convenience in the following. Then, the signal can be represented as

(10) $$s_{a}\left(K_{x},K_{y}\;;\varphi_{m}\right)=\rho \left(x,y,z\right)\cdot \;\int \int \;\exp\left\{jK_{x}x+jK_{y}y\right\}dxdy\cdot \int \exp\left(j2Kz\sin\varphi_{m}\right)dz$$

Note that the double integral in Equation (10) is the 2D Fourier transform, thus, the 2D inverse Fourier transform can be performed to both sides of (10). After the 2D inverse Fourier transform, the scattering distribution for each $\left(x,y\right)$ cell is obtained:

(11) $$s_{a}\left(x,y\;;\varphi_{m}\right)=\rho \left(x,y,z\right)\cdot \int \exp\left(j2Kz\sin\varphi_{m}\right)dz$$

Equation (11) indicates that for a determined cell $\left(x,y\right)$, the 2D imaging result is the correlation stack of the scattering coefficients of all the scattering centers with different heights *z*. Therefore, to obtain the 3D image, we need to reconstruct the height location of the scattering centers.

### 2.3. Scattering Coefficient Reconstruction along the Height Direction

**Figure 2:**
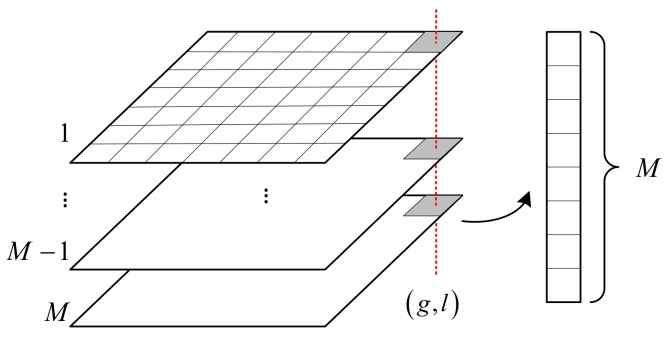
Data corresponding to the same pixel (g,l) are extracted and rearranged in a vector.

To avoid the grating lobes, the dense linear sensor array needs to satisfy the half-wavelength spacing condition. However, the wavelength of a terahertz wave is as short as less than 1 mm generally, thus, integration in practical implementation is almost impossible. Therefore, the sparse sensor array along the *Z*-axis is implemented. However, this makes the Fourier-based signal processing invalid. Considering that there are limited dominated scattering centers in the height direction, the sparse reconstruction method can provide a solution to recover the scattering coefficient distribution. The data corresponding to the same pixel cell (g,l) of the multiple 2D complex images are extracted and rearranged in a vector as the measurement data. This manipulation is shown in Figure 2.

In discrete signal processing, Equation (11) can be rewritten as

(12) $$s\left(x_{g},y_{l},\varphi_{m}\right)=\sum_{q=1}^{Q}\rho_{q}\exp\left(j2Kz_{q}\sin\varphi_{m}\right)$$

where *Q* is the number of grids divided along the height direction, and the values of the grids are $z={\left[z_{1},z_{2},\cdots ,z_{Q}\right]}^{T}$, where ${\left[\cdot \right]}^{T}$ denotes the transpose. Furthermore, Equation (12) can be presented as the vector form $s_{gl}\left(\varphi_{m}\right)=w_{m}\rho_{gl}$, where $w_{m}=\exp\left(j2K\sin\varphi_{m}z^{T}\right)\in C^{1\times Q}$, $\rho_{q}=\left[\rho_{1},\rho_{2},\cdots ,\rho_{Q}\right]$, and $\rho_{q}$ represent the scattering coefficients of the targets at grids z to be recovered. Therefore, for all $\varphi_{m}$, we have $s_{gl}\left(\varphi_{m}\right)=W\rho_{gl}$, where $s_{gl}={\left[s_{gl}\left(\varphi_{1}\right),s_{gl}\left(\varphi_{2}\right),\cdots ,s_{gl}\left(\varphi_{M}\right)\right]}^{T}\in C^{M\times 1}$, $W={\left[w_{1},w_{2},\cdots ,w_{M}\right]}^{T}\in C^{M\times Q}$. Since we will reconstruct the height information pixel by pixel, the subscript g,l will be omitted for brevity in the following. With additive white Gaussian noise $N\in C^{M\times 1}$ whose mean is zero and variance is β, we obtain

(13) s=Wρ+N

**Figure 3:**
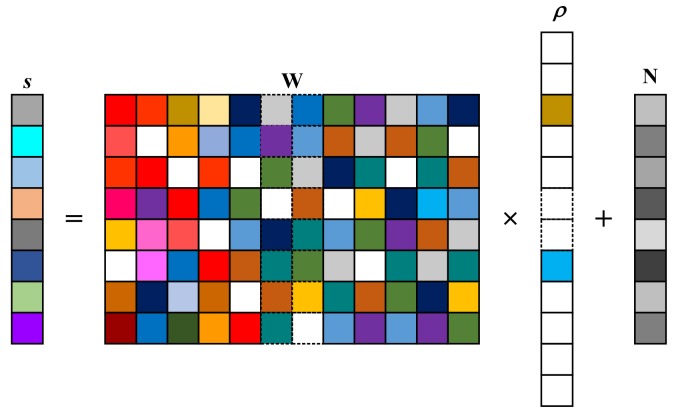
Graphical representation of Equation (13). The blank squares indicate zeros, while the colored ones indicate non-zero values.

Equation (13) can be exhibited in Figure 3.

Since Q>M, the solution of Equation (13) is underdetermined. Generally, the targets of interest in security detection behave with spatial sparsity, which means that only a few values of ρ shown in Figure 3 are meaningful. Thus, the solving of Equation (13) can be transferred to an optimization problem as

(14) $$\min_{\rho }\left|\rho \right|,s.t.∥s-W\rho ∥\leq \delta$$

where δ is a proper bound.

To solve the problem in Equation (14), sparse recovery algorithms can be adopted. In SAR imaging, the number of dominated scattering centers are generally unknown, thus, the algorithms based on the sparsity of the signal are impracticable. As analyzed in [22,23,24], sparse Bayesian learning (SBL) is an effective approach for sparse recovery. Compared with other $l_{1}$-norm minimization algorithms, the SBL-based algorithm adopts iteration during the procedure to avoid convergence to the local minimum and produces a full posterior distribution as the solution. Based on the Gaussian scale mixture (GSM), the prior probability of the distribution of scattering coefficient ρ is represented as [23]:

(15) $$p\left(\rho |\upsilon \right)=\prod_{q=1}^{Q}\frac{1}{\sqrt{2\pi \upsilon_{q}}}\exp\left\{-\frac{\rho_{q}^{2}}{2\upsilon_{q}}\right\}$$

where $\upsilon =\left[\upsilon_{1},\ldots ,\upsilon_{q},\ldots ,\upsilon_{Q}\right]$ is the hyperparameter that governs ρ.

Once the scattering coefficients are given, the probability of the measurement vector is determined by the noise, which is given by

(16) $$p\left(s|\rho ;\beta \right)={\left(\frac{1}{\sqrt{2\pi \beta }}\right)}^{M}\exp\left[-\frac{1}{2\beta }{∥s-W\rho ∥}^{2}\right]$$

With the prior probability of ρ and the conditional probability of s, the marginal probability of s is yielded by

(17) $$\begin{array}{cc} p\left(s|\upsilon ;\beta \right) & =\int p\left(s|\rho ;\beta \right)p\left(\rho |\upsilon \right)d\rho \\ & ={\left(\frac{1}{\sqrt{2\pi }}\right)}^{M}\frac{1}{\sqrt{\Sigma_{I}}}\exp\left(-\frac{1}{2}s^{T}\Sigma_{I}^{-1}s\right) \end{array}$$

where $\Sigma_{I}=\beta I+W\Lambda W^{T}$, I is the identity matrix, Λ=diag(υ).

Based on the Bayesian Theorem, the posterior probability of ρ is

(18) $$\begin{array}{cc} p\left(\rho |s,\upsilon \right) & =\frac{p\left(s|\rho ;\beta \right)p\left(\rho |\upsilon \right)}{p\left(s|\upsilon ;\beta \right)}\\ & ={\left(\frac{1}{\sqrt{2\pi }}\right)}^{Q}\frac{1}{\sqrt{\left|\Sigma_{\rho }\right|}}\exp\left[-\frac{1}{2}\rho^{T}\left(\Sigma_{\rho }^{-1}-d\cdot I\right)\rho \right] \end{array}$$

where

(19) $$\begin{array}{cc} d & =\beta^{-1}\Sigma_{\rho }W^{T}s\\ \Sigma_{\rho } & ={\left(\beta^{-1}W^{T}W+\Lambda^{-1}\right)}^{-1} \end{array}$$

From (18), we can see that the posterior probability is a Gaussian distribution with mean d and variance $\Sigma_{\rho }$, which are represented in (19). For Gaussian distribution, the mean value can be treated as the estimated scattering coefficient, i.e., the estimate $\hat{\rho }=d$. It can be seen that the estimate of the scattering coefficient is relative to the noise variance β and hyperparameter υ. For traditional SBL, β and υ can be estimated with expectation–maximization (EM) steps [22]. However, the noise variance β is a nuisance parameter and the estimate may be highly inaccurate [22]. According to [23], β can be integrated out by introducing a prior distribution, which is a gamma distribution given by

(20) $$p\left(\beta |a,b\right)=Gam\left(\beta |a,b\right)=\frac{1}{\Gamma \left(a\right)}b^{a}\beta^{a-1}\exp\left(-b\beta \right)$$

where *a* and *b* are deterministic small value, and $\Gamma \left(a\right)$ is a gamma function defined as $\Gamma \left(a\right)=\int_{0}^{\infty }u^{a-1}\exp\left(-u\right)du$. With the prior probability of β, the posterior of ρ is yielded as

(21) $$p\left(\rho |s,\upsilon \right)=\frac{\Gamma \left(a+Q\right){\left\{1+{\left(\rho -\bar{d}\right)}^{H}{\bar{\Sigma }}_{\rho }^{-1}\left(\rho -\bar{d}\right)/b\right\}}^{-\left(a+L\right)}}{\Gamma \left(a\right){\left(\pi b\right)}^{Q}\left|\bar{\Sigma }\right|}$$

The posterior distribution given by (21) is a multivariate complex Student’s t-distribution, whose mean and covariance are given by

(22) $$\begin{array}{cc} \bar{d} & ={\bar{\Sigma }}_{\rho }W^{T}s\\ {\bar{\Sigma }}_{\rho } & ={\left(W^{T}W+\Lambda^{-1}\right)}^{-1} \end{array}$$

Like Gaussian distribution, the mean value of Student’s t-distribution can also be treated as the estimated scattering coefficient, i.e., the estimate $\hat{\rho }=\bar{d}$. We can see that the estimate is merely a function of the hyperparameter rather than the noise variance β. Therefore, in order to obtain the estimate of the scattering coefficient, we have to estimate the hyperparameter υ. Using the EM approach in SBL, υ can be obtained by maximizing the marginal probability of s, which is $\arg\max_{\upsilon }p\left(s|\upsilon \right)$. The marginal probability is yielded by

(23) $$\begin{array}{cc} p\left(s|\upsilon \right) & =\int p\left(s|\rho ;\beta \right)\cdot p\left(\rho |\upsilon \right)\cdot p\left(\beta |a,b\right)d\rho d\beta \\ & =\frac{b^{a}{\left(b+s^{H}G^{-1}s\right)}^{-\left(M+a\right)}\Gamma \left(M+a\right)}{\Gamma \left(a\right)\pi^{M}\left|G\right|} \end{array}$$

where $G=I+W\Lambda W^{H}$.

Taking the logarithm of $p\left(s|\upsilon \right)$ and neglecting the terms that are independent of hyperparameter υ, the cost function is represented as

(24) $$L\left(\upsilon \right)=-\left(M+a\right)\ln\left(s^{H}G^{-1}s\right)-\ln\left(\left|G\right|\right)$$

Differentiating $L\left(\upsilon \right)$ with respect to $\upsilon_{q}$, and setting the results to zero, we can obtain

(25) $$\upsilon_{q}=\frac{1}{\left(M+a\right){\bar{d}}_{q}/\left(b+s^{H}G^{-1}s\right)+{\bar{\sigma }}_{\left(q,q\right)}}$$

where ${\bar{d}}_{q}$ is the *q*th component of $\bar{d}$ and ${\bar{\sigma }}_{\left(q,q\right)}$ is the *q*th diagonal component of ${\bar{\Sigma }}_{\rho }$.

From Equation (25), we can see that the hyperparameter υ is a function of mean $\bar{d}$ and covariance ${\bar{\Sigma }}_{\rho }$. Meanwhile, Equation (22) indicates that $\bar{d}$ and ${\bar{\Sigma }}_{\rho }$ are functions of υ. Therefore, once the initial values of υ, *a*, and *b* are given, an iterative procedure can be implemented between (22) and (25) to obtain the estimates of $\bar{d}$, ${\bar{\Sigma }}_{\rho }$, and υ. Furthermore, the scattering coefficient estimate is obtained by $\hat{\rho }=\bar{d}$. The processing procedure of the SBL mentioned above is shown in Algorithm 1.

**Algorithm 1:** Processing procedure of sparse Bayesian learning. **Input:**  2D image vector s, matrix W;  noise parameter a,b;  initial hyperparamter υ;  stop value δ; **Output:** mean $\bar{d}$, covariance ${\bar{\Sigma }}_{\rho }$; 1: **BEGIN** 2: initialize step i=1; 3: **do** 4: Compute the estimated mean ${\bar{d}}^{\left(i\right)}$ and covariance ${\bar{\Sigma }}_{\rho }^{\left(i\right)}$ using Equation (22); 5: Update $\bar{d}={\bar{d}}^{\left(i\right)}$, ${\bar{\Sigma }}_{\rho }={\bar{\Sigma }}_{\rho }^{\left(i\right)}$ 6: Calculate hyperparameter $\upsilon_{q}^{\left(i\right)},q=1,2,\cdots ,Q$ using Equation (25); 7: i=i+1; 8: **while** 9: $\frac{∥L{\left(\upsilon \right)}^{\left(i\right)}-L{\left(\upsilon \right)}^{\left(i-1\right)}∥}{L{\left(\upsilon \right)}^{\left(i-1\right)}}>\delta$; 10: **end do** 11: **return** $\bar{d}$, ${\bar{\Sigma }}_{\rho }$. 12: **END**

**Figure 4:**
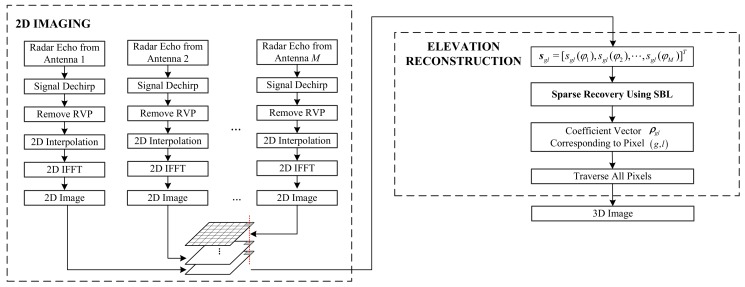
3D imaging flowchart.

Finally, combining the 2D imaging procedure with the reconstruction of the scattering coefficient along the height direction, the overall flowchart for 3D imaging is shown in Figure 4.

## 3. Simulations and Experiments

### 3.1. Simulation Results and Analysis

In this section, we present numerical simulations used to verify the effectiveness of the proposed imaging model and sparse recovery method. In the simulations, the radar center frequency is set as 340 GHz based on the atmospheric transmission window theory. A benefit pf the high center frequency is that a large bandwidth can be achieved for terahertz radar, which is set as 28 GHz here. The other main simulation system parameters are also listed in Table 1.

**Figure 5:**
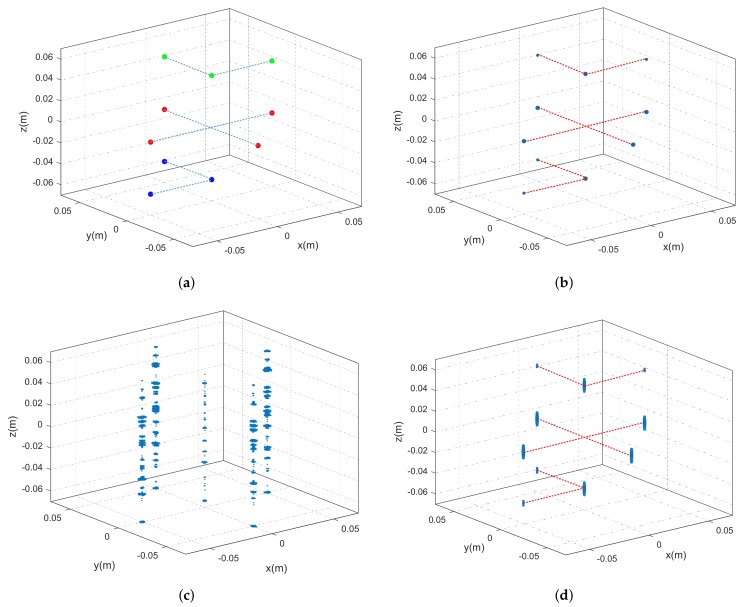
Imaging scene and 3D imaging results. Dotted lines link the points in the same plane. (**a**) Original imaging scene; (**b**) imaging result using sparse recovery with sparse sensor array; (**c**) imaging result using MF with sparse sensor array; (**d**) imaging result using MF with dense sensor array.

The simulated imaging scene is shown in Figure 5a, where there contains 10 point-scattering centers in three different planes. In Figure 5a, the points with the same color are located in the same plane. The 2D imaging method is first applied to each equivalent sensor to obtain the 2D image. After that, the sparse reconstruction algorithm is adopted to recover the scattering coefficients along the height direction for each range-azimuth cell. The final 3D imaging result using the sparse recovery method is presented in Figure 5b. Compared with Figure 5a, it can be seen that the point scattering centers are well reconstructed in the 3D space. The 3D imaging result using the MF method with sparse linear sensor array is also presented, which is shown in Figure 5c. It can be seen that the points with the same range-azimuth cell cannot be distinguished along the height direction. Moreover, the imaging result using the MF method with dense sensor array that satisfies the half-wavelength spacing condition is given in Figure 5d. It shows that the points are recovered with acceptable sidelobes.

**Figure 6:**
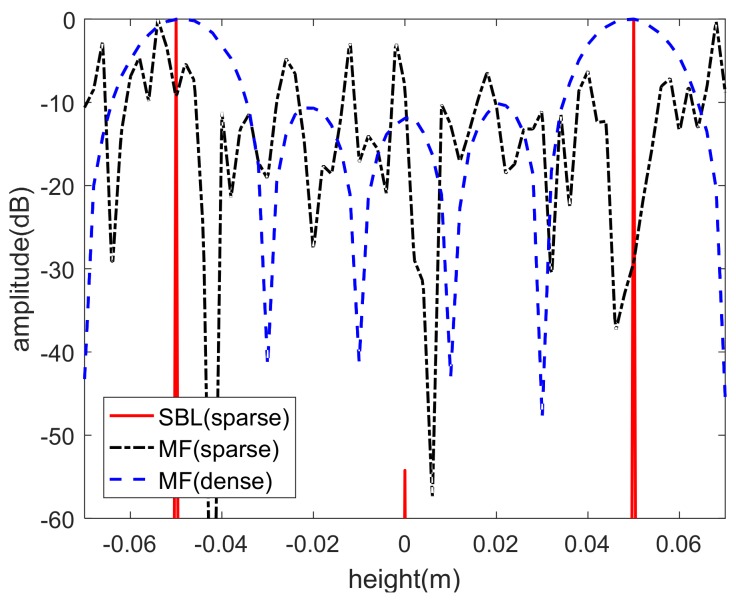
Height profile of the range-azimuth cell (0,0).

To further exhibit the imaging results, the height profiles at the range-azimuth cell (0,0) are provided in Figure 6, where the red line, black line, and blue line correspond to the imaging results of spare recovery with sparse sensor array, MF method with sparse sensor array, and MF method with dense sensor array, respectively. In the original simulated scene, there are two points in the cell (0,0) with different heights. Figure 6 shows that the blue line has two main lobes at z=−0.05 m and z=0.05 m, while the red line contains two evident peaks that conform to the main lobes. It should be declared that the sparse recovery is a super-resolution imaging method, thus the height profile has no sidelobes. On the other hand, the imaging result using MF with sparse sensor array is messy and has no prominent peaks. Therefore, the simulations demonstrate that the sparse recovery can reconstruct the height location of scattering centers with sparse linear sensor array.

### 3.2. Experiment Results and Analysis

**Figure 7:**
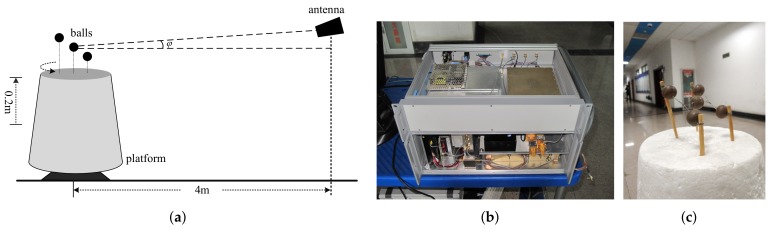
Experimental scenario. (**a**) Diagram of imaging scene; (**b**) photo of the terahertz radar system; (**c**) photo of targets.

With 0.34 THz radar, the equivalent experiment was conducted to validate the proposed imaging model and 3D imaging method. The system parameters are the same with those of the simulation shown in Table 1. The experimental scenario is presented in Figure 7. In Figure 7a, the radar is fixed while the targets are placed on a rotating platform, which is equal to the radar moving in a circular trajectory while the targets stand still. Since our radar system has no sensor array, we raise the platform step-by-step to form the relative height changing between the sensor and targets. The platform is precisely controlled to make sure that it rotates from the same original angle of each height step. Figure 7b is the optical photo of the terahertz radar with a single pair of transmitting-receiving antennas. More specified descriptions for this radar system, such as the schematic diagram and front-end setup, are presented in [8,9]. Figure 7c shows the optical photo of the targets, which contain five metallic balls in three different planes.

**Figure 8:**
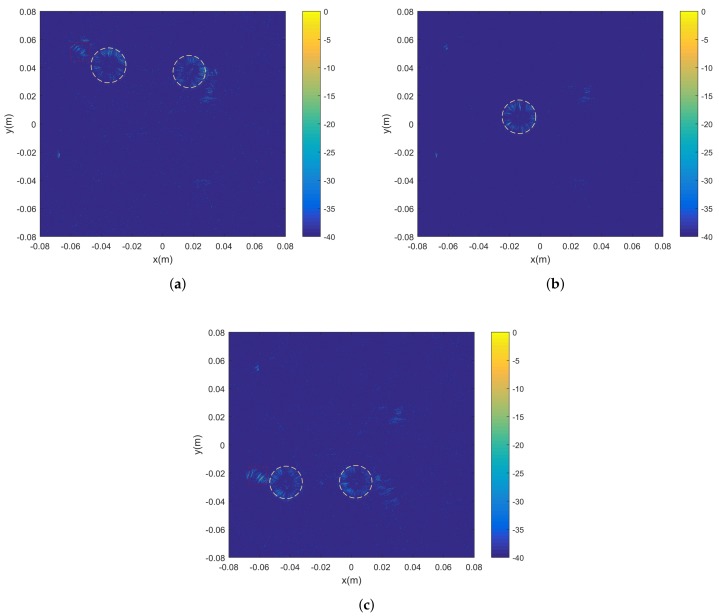
2D imaging slices of heights at (**a**) z=0.02 m; (**b**) z=0 m; (**c**) z=−0.02 m. The yellow circles mark the locations of the balls for each slice, while the regions in red squares are noise generated by the crabsticks.

The experimental results are shown in Figure 8, which contains three 2D slices corresponding to the planes, whose heights are z=0.02 m, 0 m, and −0.02 m, respectively. The yellow circles in each figure mark the locations of the metallic balls. Comparing Figure 7c with Figure 8a, we can see that only the two balls in Figure 7c, whose relative heights are in accord with the height z=0.02 m, appear remarkably in Figure 8a. Similarly, this phenomenon is also presented in Figure 8b,c. It should be noted that the regions in red squares are noise generated by the crabsticks. Therefore, the experimental results demonstrate the 3D imaging ability of the proposed imaging model and sparse recovery algorithm.

## 4. Conclusions

For the application of security detection of the human body in public areas, a 3D imaging model that combines CSAR with sparse linear sensor array was established in this study. Based on the CSAR model, $360^{{}^{\circ}}$ aspect 2D high-resolution imaging was implemented using a phase-preserving algorithm. Due to the short wavelength of terahertz waves, the sparse linear sensor array was used to form the resolution in height direction. With the obtained complex 2D images, the sparse recovery method, which is an SBL-based algorithm, was applied to obtain scattering coefficient reconstruction along the height direction. After accomplishing the reconstructions of all the range-azimuth cells, the final 3D image was obtained. Numerical simulation was performed to verify the high-resolution imaging ability of the proposed imaging model and algorithm. Furthermore, we used 0.34 THz radar to carry out the equivalent experiments, and the results demonstrate that the terahertz radar can be applied in high-resolution imaging, which indicates that terahertz radar imaging is suitable for security detection of humans in public areas. However, the imaging process described in this paper used the isotropic assumption of the targets, which may not always be true in practice, and the imaging method for aspect-dependent targets needs to be further researched.

## Figures and Tables

**Table 1:** Main system parameters

Parameters	Values
Carrier frequency	340 GHz
Bandwidth	28 GHz
Pulse duration time	1 ms
Radar radius	5 m
Equivalent receiving antenna number	9
Aperture length in height direction	0.2 m
